# [*N*′-(5-Chloro-2-oxidobenzyl­idene-κ*O*)-3-hydr­oxy-2-naphthohydrazidato-κ^2^ *N*′,*O* ^2^]diphenyl­tin(IV)

**DOI:** 10.1107/S1600536809050107

**Published:** 2009-11-28

**Authors:** See Mun Lee, Kong Mun Lo, Hapipah Mohd Ali, Seik Weng Ng

**Affiliations:** a Department of Chemistry, University of Malaya, 50603 Kuala Lumpur, Malaysia

## Abstract

The Sn^IV^ atom in the title compound, [Sn(C_6_H_5_)_2_(C_18_H_11_ClN_2_O_3_)], is *O*,*N*,*O*′-chelated by the deprotonated Schiff base ligand and further bonded by two phenyl rings in a distorted *cis*-C_2_SnNO_2_ trigonal-bipyramidal geometry [C—Sn—C = 125.7 (2)°]. The two phenyl rings are oriented at a dihedral angle of 55.2 (3)°. Intra­molecular O—H⋯N hydrogen bonding is present in the crystal structure.

## Related literature

For the Sn(CH_3_)_2_(C_18_H_11_ClN_2_O_3_) analog, see: Lee *et al.* (2009 ▶).
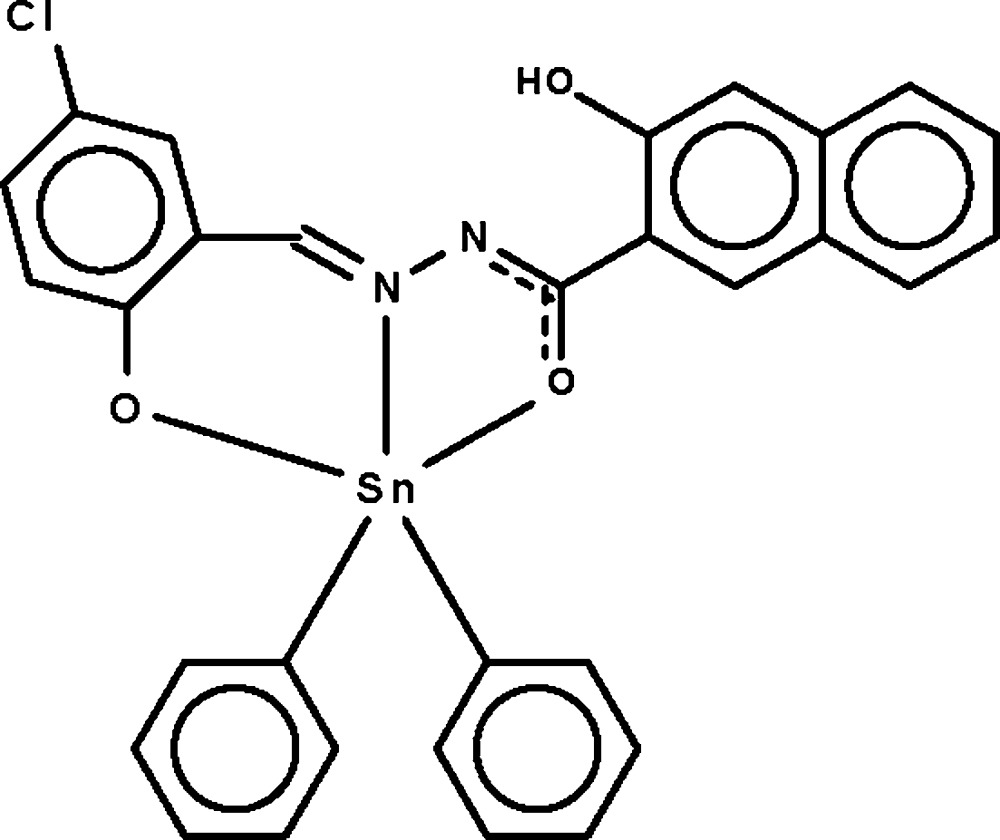


## Experimental

### 

#### Crystal data

[Sn(C_6_H_5_)_2_(C_18_H_11_ClN_2_O_3_)]
*M* *_r_* = 611.63
Triclinic, 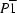

*a* = 10.5690 (4) Å
*b* = 10.9788 (4) Å
*c* = 11.8319 (4) Å
α = 68.381 (2)°
β = 82.450 (2)°
γ = 82.672 (2)°
*V* = 1260.60 (8) Å^3^
*Z* = 2
Mo *K*α radiation
μ = 1.16 mm^−1^
*T* = 140 K
0.30 × 0.25 × 0.20 mm

#### Data collection

Bruker SMART APEX diffractometer
Absorption correction: multi-scan (*SADABS*; Sheldrick, 1996 ▶) *T*_min_ = 0.798, *T*_max_ = 1.000
6070 measured reflections
4280 independent reflections
3563 reflections with *I* > 2σ(*I*)
*R*_int_ = 0.026

#### Refinement

*R*[*F* ^2^ > 2σ(*F* ^2^)] = 0.043
*wR*(*F* ^2^) = 0.126
*S* = 1.05
4280 reflections
338 parameters
1 restraint
H atoms treated by a mixture of independent and constrained refinement
Δρ_max_ = 1.39 e Å^−3^
Δρ_min_ = −0.70 e Å^−3^

### 

Data collection: *APEX2* (Bruker, 2008 ▶); cell refinement: *SAINT* (Bruker, 2008 ▶); data reduction: *SAINT*; program(s) used to solve structure: *SHELXS97* (Sheldrick, 2008 ▶); program(s) used to refine structure: *SHELXL97* (Sheldrick, 2008 ▶); molecular graphics: *X-SEED* (Barbour, 2001 ▶); software used to prepare material for publication: *publCIF* (Westrip, 2009 ▶).

## Supplementary Material

Crystal structure: contains datablocks global, I. DOI: 10.1107/S1600536809050107/xu2687sup1.cif

Structure factors: contains datablocks I. DOI: 10.1107/S1600536809050107/xu2687Isup2.hkl

Additional supplementary materials:  crystallographic information; 3D view; checkCIF report

## Figures and Tables

**Table 1:** Selected bond lengths (Å)

Sn1—O1	2.057 (4)
Sn1—O2	2.150 (3)
Sn1—N1	2.166 (4)
Sn1—C1	2.131 (5)
Sn1—C7	2.124 (5)

**Table 2:** Hydrogen-bond geometry (Å, °)

*D*—H⋯*A*	*D*—H	H⋯*A*	*D*⋯*A*	*D*—H⋯*A*
O3—H3⋯N2	0.838 (10)	1.90 (4)	2.622 (6)	144 (6)

**Fig. 1.**
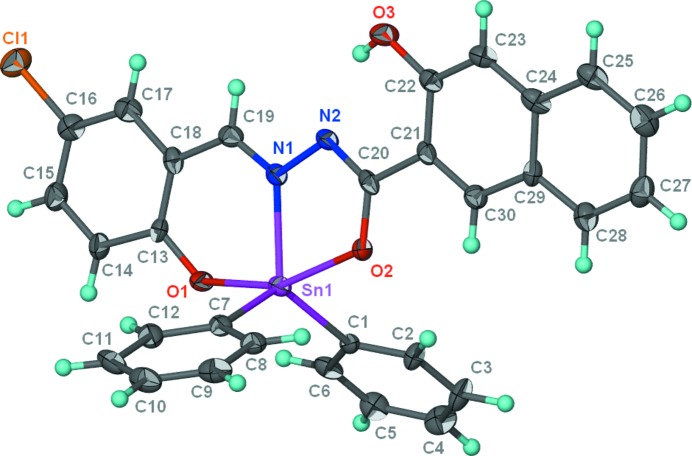
Thermal ellipsoid plot (Barbour, 2001) of Sn(C6H5)2(C18H11ClN2O3) at the 70% probability level; hydrogen atoms are drawn as spheres of arbitrary radius.

## supplementary crystallographic information

### Experimental

The Schiff base was prepared by the condensation of 3-hydroxy-2-naphthoylhydrazide and 5-chlorobenzaldehyde. The Schiff base (0.5 g, 1.5 mmol) and diphenyltin dichloride (0.52 g, 1.5 mmol) in a mixture (1:1) of methanol/chloroform was heated for 1 h. The filtered solution yielded the yellow crystals when allowed to evaporate slowly.

### Refinement

Carbon-bound H-atoms were placed in calculated positions (C–H 0.95 Å) and were included in the refinement in the riding model approximation, with *U*(H) set to 1.2*U*(C). The hydroxy H-atom was located in a difference Fourier map and was refined with a distance restraint of O–H 0.84±0.01 Å.

The final difference Fourier map had a peak in the vicinity of Sn1.

### Crystal data

**Table:**

[Sn(C_6_H_5_)_2_(C_18_H_11_ClN_2_O_3_)]	*Z* = 2
*M**_r_* = 611.63	*F*(000) = 612
Triclinic, *P*1	*D*_x_ = 1.611 Mg m^−^^3^
Hall symbol: -P 1	Mo *K*α radiation, λ = 0.71073 Å
*a* = 10.5690 (4) Å	Cell parameters from 2361 reflections
*b* = 10.9788 (4) Å	θ = 2.5–26.8°
*c* = 11.8319 (4) Å	µ = 1.16 mm^−^^1^
α = 68.381 (2)°	*T* = 140 K
β = 82.450 (2)°	Block, yellow
γ = 82.672 (2)°	0.30 × 0.25 × 0.20 mm
*V* = 1260.60 (8) Å^3^	

### Data collection

**Table:**

Bruker SMART APEX diffractometer	4280 independent reflections
Radiation source: fine-focus sealed tube	3563 reflections with *I* > 2σ(*I*)
graphite	*R*_int_ = 0.026
ω scans	θ_max_ = 25.0°, θ_min_ = 1.9°
Absorption correction: multi-scan (*SADABS*; Sheldrick, 1996)	*h* = −9→12
*T*_min_ = 0.798, *T*_max_ = 1.000	*k* = −12→13
6070 measured reflections	*l* = −14→14

### Refinement

**Table:**

Refinement on *F*^2^	Primary atom site location: structure-invariant direct methods
Least-squares matrix: full	Secondary atom site location: difference Fourier map
*R*[*F*^2^ > 2σ(*F*^2^)] = 0.043	Hydrogen site location: inferred from neighbouring sites
*wR*(*F*^2^) = 0.126	H atoms treated by a mixture of independent and constrained refinement
*S* = 1.05	*w* = 1/[σ^2^(*F*_o_^2^) + (0.0736*P*)^2^ + 1.6275*P*] where *P* = (*F*_o_^2^ + 2*F*_c_^2^)/3
4280 reflections	(Δ/σ)_max_ = 0.001
338 parameters	Δρ_max_ = 1.39 e Å^−^^3^
1 restraint	Δρ_min_ = −0.70 e Å^−^^3^

### Fractional atomic coordinates and isotropic or equivalent isotropic displacement parameters (Å^2^)

**Table:**

	*x*	*y*	*z*	*U*_iso_*/*U*_eq_	
Sn1	0.70294 (4)	0.27373 (3)	0.41404 (3)	0.02376 (15)	
Cl1	0.26756 (16)	0.83455 (15)	0.05708 (14)	0.0421 (4)	
O1	0.5939 (4)	0.3459 (4)	0.2678 (4)	0.0301 (9)	
O2	0.8166 (3)	0.2790 (4)	0.5498 (3)	0.0260 (8)	
O3	0.8095 (4)	0.6332 (4)	0.6156 (4)	0.0342 (9)	
H3	0.785 (6)	0.626 (6)	0.554 (4)	0.036 (18)*	
N1	0.6825 (4)	0.4749 (4)	0.4076 (4)	0.0227 (9)	
N2	0.7518 (4)	0.5023 (4)	0.4861 (4)	0.0243 (10)	
C1	0.8655 (5)	0.1998 (5)	0.3241 (5)	0.0239 (11)	
C2	0.9726 (5)	0.1487 (5)	0.3839 (5)	0.0280 (12)	
H2	0.9745	0.1478	0.4642	0.034*	
C3	1.0794 (6)	0.0977 (6)	0.3278 (5)	0.0376 (15)	
H3A	1.1540	0.0622	0.3698	0.045*	
C4	1.0764 (6)	0.0992 (6)	0.2115 (6)	0.0399 (15)	
H4	1.1484	0.0628	0.1738	0.048*	
C5	0.9670 (6)	0.1543 (6)	0.1480 (5)	0.0346 (14)	
H5	0.9659	0.1569	0.0670	0.041*	
C6	0.8606 (6)	0.2049 (5)	0.2042 (5)	0.0306 (13)	
H6	0.7861	0.2422	0.1624	0.037*	
C7	0.5649 (5)	0.1587 (5)	0.5428 (5)	0.0256 (12)	
C8	0.5989 (6)	0.0795 (5)	0.6585 (5)	0.0297 (13)	
H8	0.6825	0.0785	0.6804	0.036*	
C9	0.5098 (6)	0.0019 (6)	0.7421 (6)	0.0396 (15)	
H9	0.5329	−0.0526	0.8211	0.047*	
C10	0.3894 (6)	0.0032 (6)	0.7117 (6)	0.0403 (16)	
H10	0.3294	−0.0505	0.7697	0.048*	
C11	0.3539 (6)	0.0823 (6)	0.5971 (6)	0.0415 (16)	
H11	0.2695	0.0847	0.5765	0.050*	
C12	0.4438 (5)	0.1581 (6)	0.5126 (6)	0.0314 (13)	
H12	0.4212	0.2102	0.4328	0.038*	
C13	0.5173 (5)	0.4566 (5)	0.2272 (5)	0.0227 (11)	
C14	0.4279 (5)	0.4645 (6)	0.1468 (5)	0.0297 (12)	
H14	0.4196	0.3894	0.1275	0.036*	
C15	0.3527 (6)	0.5769 (6)	0.0957 (5)	0.0336 (13)	
H15	0.2928	0.5800	0.0413	0.040*	
C16	0.3640 (6)	0.6880 (6)	0.1234 (5)	0.0311 (13)	
C17	0.4479 (6)	0.6835 (6)	0.2032 (5)	0.0307 (13)	
H17	0.4539	0.7593	0.2221	0.037*	
C18	0.5258 (5)	0.5677 (5)	0.2580 (5)	0.0244 (11)	
C19	0.6084 (5)	0.5730 (5)	0.3425 (5)	0.0266 (12)	
H19	0.6091	0.6551	0.3520	0.032*	
C20	0.8181 (5)	0.3960 (5)	0.5544 (5)	0.0247 (12)	
C21	0.8990 (5)	0.4095 (5)	0.6409 (5)	0.0235 (11)	
C22	0.8891 (5)	0.5257 (5)	0.6705 (5)	0.0248 (11)	
C23	0.9596 (5)	0.5304 (5)	0.7572 (5)	0.0286 (12)	
H23	0.9485	0.6059	0.7798	0.034*	
C24	1.0496 (6)	0.4240 (5)	0.8146 (5)	0.0296 (13)	
C25	1.1290 (6)	0.4294 (6)	0.8994 (5)	0.0338 (13)	
H25	1.1199	0.5042	0.9229	0.041*	
C26	1.2191 (6)	0.3278 (6)	0.9484 (5)	0.0384 (15)	
H26	1.2726	0.3337	1.0044	0.046*	
C27	1.2331 (6)	0.2150 (6)	0.9166 (5)	0.0353 (14)	
H27	1.2963	0.1455	0.9501	0.042*	
C28	1.1540 (5)	0.2065 (6)	0.8362 (5)	0.0307 (13)	
H28	1.1618	0.1291	0.8166	0.037*	
C29	1.0625 (5)	0.3094 (5)	0.7826 (5)	0.0244 (11)	
C30	0.9820 (5)	0.3049 (5)	0.6981 (5)	0.0264 (12)	
H30	0.9856	0.2268	0.6804	0.032*	

### Atomic displacement parameters (Å^2^)

**Table:**

	*U*^11^	*U*^22^	*U*^33^	*U*^12^	*U*^13^	*U*^23^
Sn1	0.0256 (2)	0.0222 (2)	0.0264 (2)	−0.00428 (14)	−0.00185 (14)	−0.01152 (15)
Cl1	0.0500 (10)	0.0377 (8)	0.0378 (8)	0.0106 (7)	−0.0166 (7)	−0.0129 (7)
O1	0.034 (2)	0.026 (2)	0.040 (2)	0.0033 (17)	−0.0167 (18)	−0.0194 (17)
O2	0.024 (2)	0.0249 (19)	0.033 (2)	0.0014 (15)	−0.0118 (16)	−0.0122 (16)
O3	0.046 (3)	0.027 (2)	0.035 (2)	0.0013 (19)	−0.0153 (19)	−0.0158 (18)
N1	0.022 (2)	0.025 (2)	0.023 (2)	−0.0039 (18)	−0.0017 (18)	−0.0104 (19)
N2	0.026 (2)	0.024 (2)	0.027 (2)	−0.0042 (19)	−0.0062 (19)	−0.0121 (19)
C1	0.015 (3)	0.020 (3)	0.037 (3)	−0.004 (2)	0.003 (2)	−0.011 (2)
C2	0.020 (3)	0.031 (3)	0.028 (3)	0.004 (2)	−0.004 (2)	−0.007 (2)
C3	0.026 (3)	0.042 (4)	0.036 (3)	0.011 (3)	−0.007 (3)	−0.006 (3)
C4	0.041 (4)	0.040 (3)	0.035 (3)	−0.002 (3)	0.010 (3)	−0.014 (3)
C5	0.039 (4)	0.037 (3)	0.027 (3)	−0.008 (3)	0.001 (3)	−0.011 (3)
C6	0.038 (3)	0.026 (3)	0.030 (3)	−0.014 (2)	0.003 (2)	−0.011 (2)
C7	0.022 (3)	0.028 (3)	0.032 (3)	−0.003 (2)	0.005 (2)	−0.019 (2)
C8	0.030 (3)	0.027 (3)	0.036 (3)	−0.002 (2)	0.001 (2)	−0.017 (2)
C9	0.049 (4)	0.030 (3)	0.040 (4)	−0.005 (3)	0.006 (3)	−0.016 (3)
C10	0.039 (4)	0.037 (3)	0.050 (4)	−0.017 (3)	0.017 (3)	−0.024 (3)
C11	0.033 (4)	0.045 (4)	0.062 (4)	−0.017 (3)	0.012 (3)	−0.038 (3)
C12	0.027 (3)	0.035 (3)	0.038 (3)	−0.002 (2)	0.000 (2)	−0.022 (3)
C13	0.014 (3)	0.027 (3)	0.025 (3)	0.002 (2)	−0.002 (2)	−0.009 (2)
C14	0.024 (3)	0.034 (3)	0.034 (3)	0.001 (2)	−0.010 (2)	−0.014 (3)
C15	0.030 (3)	0.035 (3)	0.033 (3)	0.002 (3)	−0.013 (2)	−0.007 (3)
C16	0.032 (3)	0.031 (3)	0.026 (3)	−0.003 (3)	0.003 (2)	−0.008 (2)
C17	0.035 (3)	0.033 (3)	0.027 (3)	−0.005 (3)	0.002 (2)	−0.015 (2)
C18	0.019 (3)	0.033 (3)	0.020 (3)	−0.006 (2)	0.000 (2)	−0.008 (2)
C19	0.028 (3)	0.027 (3)	0.028 (3)	−0.004 (2)	0.001 (2)	−0.014 (2)
C20	0.018 (3)	0.035 (3)	0.026 (3)	−0.011 (2)	0.005 (2)	−0.016 (2)
C21	0.017 (3)	0.029 (3)	0.027 (3)	−0.008 (2)	0.001 (2)	−0.012 (2)
C22	0.025 (3)	0.026 (3)	0.024 (3)	−0.008 (2)	0.001 (2)	−0.009 (2)
C23	0.036 (3)	0.025 (3)	0.027 (3)	−0.007 (2)	0.000 (2)	−0.011 (2)
C24	0.041 (3)	0.030 (3)	0.019 (3)	−0.016 (3)	0.003 (2)	−0.007 (2)
C25	0.044 (4)	0.033 (3)	0.026 (3)	−0.015 (3)	0.003 (3)	−0.010 (2)
C26	0.042 (4)	0.044 (4)	0.028 (3)	−0.012 (3)	−0.007 (3)	−0.007 (3)
C27	0.028 (3)	0.035 (3)	0.031 (3)	0.001 (3)	−0.006 (2)	0.001 (3)
C28	0.027 (3)	0.038 (3)	0.027 (3)	−0.006 (2)	−0.001 (2)	−0.011 (2)
C29	0.016 (3)	0.030 (3)	0.023 (3)	−0.001 (2)	−0.002 (2)	−0.005 (2)
C30	0.026 (3)	0.030 (3)	0.026 (3)	−0.003 (2)	−0.003 (2)	−0.013 (2)

### Geometric parameters (Å, °)

**Table:**

Sn1—O1	2.057 (4)	C11—C12	1.391 (8)
Sn1—O2	2.150 (3)	C11—H11	0.9500
Sn1—N1	2.166 (4)	C12—H12	0.9500
Sn1—C1	2.131 (5)	C13—C14	1.400 (7)
Sn1—C7	2.124 (5)	C13—C18	1.411 (8)
Cl1—C16	1.763 (6)	C14—C15	1.359 (8)
O1—C13	1.336 (6)	C14—H14	0.9500
O2—C20	1.308 (6)	C15—C16	1.397 (8)
O3—C22	1.360 (7)	C15—H15	0.9500
O3—H3	0.838 (10)	C16—C17	1.361 (8)
N1—C19	1.304 (7)	C17—C18	1.407 (8)
N1—N2	1.389 (6)	C17—H17	0.9500
N2—C20	1.323 (7)	C18—C19	1.433 (8)
C1—C2	1.361 (7)	C19—H19	0.9500
C1—C6	1.407 (8)	C20—C21	1.475 (7)
C2—C3	1.394 (8)	C21—C30	1.367 (8)
C2—H2	0.9500	C21—C22	1.431 (7)
C3—C4	1.375 (9)	C22—C23	1.364 (8)
C3—H3A	0.9500	C23—C24	1.426 (8)
C4—C5	1.408 (9)	C23—H23	0.9500
C4—H4	0.9500	C24—C25	1.411 (8)
C5—C6	1.389 (8)	C24—C29	1.427 (8)
C5—H5	0.9500	C25—C26	1.373 (9)
C6—H6	0.9500	C25—H25	0.9500
C7—C12	1.375 (8)	C26—C27	1.406 (9)
C7—C8	1.390 (8)	C26—H26	0.9500
C8—C9	1.389 (8)	C27—C28	1.381 (8)
C8—H8	0.9500	C27—H27	0.9500
C9—C10	1.364 (9)	C28—C29	1.404 (8)
C9—H9	0.9500	C28—H28	0.9500
C10—C11	1.385 (10)	C29—C30	1.413 (7)
C10—H10	0.9500	C30—H30	0.9500
			
O1—Sn1—C7	97.47 (19)	O1—C13—C18	123.0 (5)
O1—Sn1—C1	96.68 (18)	C14—C13—C18	118.5 (5)
C7—Sn1—C1	125.67 (19)	C15—C14—C13	121.6 (5)
O1—Sn1—O2	157.66 (14)	C15—C14—H14	119.2
C7—Sn1—O2	94.59 (18)	C13—C14—H14	119.2
C1—Sn1—O2	91.41 (18)	C14—C15—C16	119.7 (5)
O1—Sn1—N1	84.68 (15)	C14—C15—H15	120.2
C7—Sn1—N1	111.96 (17)	C16—C15—H15	120.2
C1—Sn1—N1	121.43 (17)	C17—C16—C15	120.5 (5)
O2—Sn1—N1	73.37 (15)	C17—C16—Cl1	119.4 (4)
C13—O1—Sn1	131.1 (3)	C15—C16—Cl1	120.1 (5)
C20—O2—Sn1	114.2 (3)	C16—C17—C18	120.7 (5)
C22—O3—H3	109 (4)	C16—C17—H17	119.6
C19—N1—N2	115.6 (4)	C18—C17—H17	119.6
C19—N1—Sn1	127.8 (4)	C17—C18—C13	118.9 (5)
N2—N1—Sn1	116.6 (3)	C17—C18—C19	116.0 (5)
C20—N2—N1	111.8 (4)	C13—C18—C19	125.1 (5)
C2—C1—C6	121.1 (5)	N1—C19—C18	125.7 (5)
C2—C1—Sn1	119.2 (4)	N1—C19—H19	117.2
C6—C1—Sn1	119.6 (4)	C18—C19—H19	117.2
C1—C2—C3	120.3 (5)	O2—C20—N2	123.9 (5)
C1—C2—H2	119.8	O2—C20—C21	117.6 (5)
C3—C2—H2	119.8	N2—C20—C21	118.4 (5)
C4—C3—C2	119.7 (6)	C30—C21—C22	119.4 (5)
C4—C3—H3A	120.1	C30—C21—C20	118.5 (5)
C2—C3—H3A	120.1	C22—C21—C20	122.0 (5)
C3—C4—C5	120.3 (6)	O3—C22—C23	118.0 (5)
C3—C4—H4	119.8	O3—C22—C21	122.0 (5)
C5—C4—H4	119.8	C23—C22—C21	120.0 (5)
C6—C5—C4	119.8 (6)	C22—C23—C24	121.2 (5)
C6—C5—H5	120.1	C22—C23—H23	119.4
C4—C5—H5	120.1	C24—C23—H23	119.4
C5—C6—C1	118.6 (6)	C25—C24—C23	122.3 (5)
C5—C6—H6	120.7	C25—C24—C29	118.8 (5)
C1—C6—H6	120.7	C23—C24—C29	118.8 (5)
C12—C7—C8	119.4 (5)	C26—C25—C24	120.8 (6)
C12—C7—Sn1	121.2 (4)	C26—C25—H25	119.6
C8—C7—Sn1	119.4 (4)	C24—C25—H25	119.6
C9—C8—C7	119.5 (6)	C25—C26—C27	120.7 (6)
C9—C8—H8	120.2	C25—C26—H26	119.6
C7—C8—H8	120.2	C27—C26—H26	119.6
C10—C9—C8	120.6 (6)	C28—C27—C26	119.3 (5)
C10—C9—H9	119.7	C28—C27—H27	120.3
C8—C9—H9	119.7	C26—C27—H27	120.3
C9—C10—C11	120.5 (6)	C27—C28—C29	121.5 (6)
C9—C10—H10	119.8	C27—C28—H28	119.3
C11—C10—H10	119.8	C29—C28—H28	119.3
C10—C11—C12	118.9 (6)	C28—C29—C30	122.9 (5)
C10—C11—H11	120.5	C28—C29—C24	118.8 (5)
C12—C11—H11	120.5	C30—C29—C24	118.3 (5)
C7—C12—C11	121.0 (6)	C21—C30—C29	122.1 (5)
C7—C12—H12	119.5	C21—C30—H30	119.0
C11—C12—H12	119.5	C29—C30—H30	119.0
O1—C13—C14	118.4 (5)		
			
C7—Sn1—O1—C13	92.8 (5)	Sn1—O1—C13—C14	−162.6 (4)
C1—Sn1—O1—C13	−139.8 (5)	Sn1—O1—C13—C18	20.0 (8)
O2—Sn1—O1—C13	−29.3 (7)	O1—C13—C14—C15	−175.7 (5)
N1—Sn1—O1—C13	−18.7 (5)	C18—C13—C14—C15	1.9 (8)
O1—Sn1—O2—C20	8.6 (6)	C13—C14—C15—C16	0.0 (9)
C7—Sn1—O2—C20	−113.9 (4)	C14—C15—C16—C17	−1.4 (9)
C1—Sn1—O2—C20	120.1 (4)	C14—C15—C16—Cl1	179.7 (5)
N1—Sn1—O2—C20	−2.3 (3)	C15—C16—C17—C18	0.8 (8)
O1—Sn1—N1—C19	9.4 (4)	Cl1—C16—C17—C18	179.8 (4)
C7—Sn1—N1—C19	−86.5 (5)	C16—C17—C18—C13	1.1 (8)
C1—Sn1—N1—C19	104.0 (5)	C16—C17—C18—C19	−178.6 (5)
O2—Sn1—N1—C19	−174.8 (5)	O1—C13—C18—C17	175.0 (5)
O1—Sn1—N1—N2	−173.9 (3)	C14—C13—C18—C17	−2.4 (8)
C7—Sn1—N1—N2	90.2 (4)	O1—C13—C18—C19	−5.2 (8)
C1—Sn1—N1—N2	−79.3 (4)	C14—C13—C18—C19	177.3 (5)
O2—Sn1—N1—N2	1.9 (3)	N2—N1—C19—C18	−178.3 (5)
C19—N1—N2—C20	175.9 (5)	Sn1—N1—C19—C18	−1.6 (8)
Sn1—N1—N2—C20	−1.2 (5)	C17—C18—C19—N1	176.1 (5)
O1—Sn1—C1—C2	175.0 (4)	C13—C18—C19—N1	−3.7 (9)
C7—Sn1—C1—C2	−80.8 (5)	Sn1—O2—C20—N2	2.8 (6)
O2—Sn1—C1—C2	15.9 (4)	Sn1—O2—C20—C21	−177.0 (3)
N1—Sn1—C1—C2	87.2 (5)	N1—N2—C20—O2	−1.1 (7)
O1—Sn1—C1—C6	−4.8 (4)	N1—N2—C20—C21	178.7 (4)
C7—Sn1—C1—C6	99.4 (4)	O2—C20—C21—C30	9.5 (7)
O2—Sn1—C1—C6	−163.9 (4)	N2—C20—C21—C30	−170.4 (5)
N1—Sn1—C1—C6	−92.6 (4)	O2—C20—C21—C22	−167.7 (5)
C6—C1—C2—C3	−1.3 (8)	N2—C20—C21—C22	12.5 (7)
Sn1—C1—C2—C3	178.9 (4)	C30—C21—C22—O3	179.2 (5)
C1—C2—C3—C4	0.0 (9)	C20—C21—C22—O3	−3.7 (8)
C2—C3—C4—C5	1.3 (9)	C30—C21—C22—C23	−1.6 (8)
C3—C4—C5—C6	−1.4 (9)	C20—C21—C22—C23	175.5 (5)
C4—C5—C6—C1	0.2 (8)	O3—C22—C23—C24	−177.0 (5)
C2—C1—C6—C5	1.2 (8)	C21—C22—C23—C24	3.8 (8)
Sn1—C1—C6—C5	−179.0 (4)	C22—C23—C24—C25	176.3 (5)
O1—Sn1—C7—C12	−3.0 (4)	C22—C23—C24—C29	−1.9 (8)
C1—Sn1—C7—C12	−106.8 (4)	C23—C24—C25—C26	−176.8 (5)
O2—Sn1—C7—C12	158.1 (4)	C29—C24—C25—C26	1.5 (8)
N1—Sn1—C7—C12	84.2 (4)	C24—C25—C26—C27	−1.0 (9)
O1—Sn1—C7—C8	175.0 (4)	C25—C26—C27—C28	−0.7 (9)
C1—Sn1—C7—C8	71.2 (5)	C26—C27—C28—C29	1.9 (9)
O2—Sn1—C7—C8	−23.8 (4)	C27—C28—C29—C30	178.6 (5)
N1—Sn1—C7—C8	−97.7 (4)	C27—C28—C29—C24	−1.4 (8)
C12—C7—C8—C9	−0.5 (8)	C25—C24—C29—C28	−0.3 (8)
Sn1—C7—C8—C9	−178.6 (4)	C23—C24—C29—C28	178.0 (5)
C7—C8—C9—C10	−0.3 (8)	C25—C24—C29—C30	179.7 (5)
C8—C9—C10—C11	−0.2 (9)	C23—C24—C29—C30	−2.0 (7)
C9—C10—C11—C12	1.4 (9)	C22—C21—C30—C29	−2.5 (8)
C8—C7—C12—C11	1.8 (8)	C20—C21—C30—C29	−179.7 (5)
Sn1—C7—C12—C11	179.8 (4)	C28—C29—C30—C21	−175.7 (5)
C10—C11—C12—C7	−2.2 (8)	C24—C29—C30—C21	4.3 (8)

### Hydrogen-bond geometry (Å, °)

**Table:**

*D*—H···*A*	*D*—H	H···*A*	*D*···*A*	*D*—H···*A*
O3—H3···N2	0.84 (1)	1.90 (4)	2.622 (6)	144 (6)

## References

[bb1] Barbour, L. J. (2001). *J. Supramol. Chem.* **1**, 189–191.

[bb2] Bruker (2008). *APEX2* and *SAINT*. Bruker AXS Inc., Madison, Wisconsin, USA.

[bb3] Lee, S. M., Lo, K. M., Mohd Ali, H. & Ng, S. W. (2009). *Acta Cryst.* E**65**, m816. 10.1107/S1600536809022259 PMC2969223 21582739

[bb4] Sheldrick, G. M. (1996). *SADABS*. University of Göttingen, Germany.

[bb5] Sheldrick, G. M. (2008). *Acta Cryst.* A**64**, 112–122. 10.1107/S0108767307043930 18156677

[bb6] Westrip, S. P. (2009). *publCIF*. In preparation.

